# Health status and health services utilization in elderly Koreans

**DOI:** 10.1186/s12939-014-0073-7

**Published:** 2014-08-27

**Authors:** Ju Moon Park

**Affiliations:** Department of Urban Policy and Administration, Incheon National University, 119 Academy-ro, Yeonsu-gu, 402-750 Incheon, South Korea

**Keywords:** Health status, Health services utilization, Elderly Koreans

## Abstract

**Background:**

Korea is aging faster than any other country as the ‘baby boomers’ grow older. The purpose of this study is to describe the health status and health services utilization of older Koreans and examine the factors that are associated with effective health services utilization.

**Methods:**

Based on the 2008 Korean Longitudinal Study of Ageing which was conducted with Korean men and women aged 45 years or older, descriptive and logistic regression analysis was performed. The sample for this study was 4040 individuals who indicated they were 65 years or older. Bivariate analyses (chi-square tests) were used to examine the differences between men and women. Logistic regression analyses were then used to determine factors significantly associated with health services utilization.

**Results:**

More women (29.3%) than men (14.5%) rated their health as poor or very poor. A significantly higher number of women than men reported having hypertension, arthritis or heart disease, while a larger number of men reported having cancer or lung problems. Age, education, income, and presence of chronic conditions significantly predicted the residents’ self-rated health. Respondents with chronic conditions were twice as likely to report self-rated poor health (Odds Ratio: 2.26, 95% CI: 1.91-2.67) than residents with no chronic conditions. Those who were more likely to have used primary care services, such as a physician or traditional Korean medicine, included those 80 and older, men, those who had a chronic condition or poor health status, and lower-income individuals.

**Conclusions:**

Respondents with poor health status were significantly older, less-educated, poorer and had a higher rate of chronic conditions. Health-related need factors and income were important predictors of Korean elders using physician services and/or traditional Korean medicine. This study provides an important contribution to the knowledge base of Korean elders. The findings show that elders in poor health status were significantly older and poorer, with higher rates of chronic conditions and health services utilization, which should help in the health care planning required to address this issue.

## Background

In the Republic of Korea (hereafter Korea), the entry of baby-boomers into old age is a social policy issue. This is because Korea is aging faster than any other country as the ‘baby boomers’ grow older. In fact, the speed of aging in Korea is unprecedented in human history [1], taking only 18 years to double the aged population from 7 – 14% (least number of years) [2], overtaking even Japan. The percentage of the elderly aged 65 and above increased from 7.2% in 2000 to 11.3% in 2010, and it is expected to rise to 14.0% by 2017 and 37.4% in 2050 [3].

From a health perspective, rapid aging means that the population’s health burden is likely to worsen, as older people tend to spend a greater proportion on health care than other sectors of the population, and will continue to require such a high level of spending for their health care needs in the future. This implies that the Korean health care system needs to be reformed so as to be able to provide sufficient care for the increasing needs of the elderly [4].

Despite the fact that aging is frequently accompanied by an increase in chronic conditions and disabilities, there has been a paucity of reliable and/or representative data on the health-related issues of Korean elderly [5-7]. Among the few useful studies that have been conducted is a Korean Longitudinal Study of Aging, which was conducted with Korean men and women aged 45 years and older. Some of the most prevalent chronic diseases were found to be arthritis, hypertension, osteoporosis, and diabetes. The pain suffered from arthritis negatively affects the overall quality of life, and the health care costs for hypertension and diabetes were ranked the first and the third highest in Korea, respectively [6,7]. Both the visible and hidden costs of such chronic conditions are largely borne by the elderly themselves, although various groups have argued that society should also bear the cost [8].

Even though general health in Korea reportedly continues to improve, improvements in the quality of health of older people is lagging. The subjective health status of adults overall has become better, while the subjective health status among the elderly has worsened. This is evident when the decline in the health status of the elderly is compared with the health status of young and middle-aged adults, which has improved substantially [4]. The subjective health status of the elderly warrants more attention from policy makers and researchers, because they have a variety of chronic conditions that lead to their lower health status [9].

Changing disease patterns and the increase in the need for chronic disease management have increased interest in the use of traditional Korean medicine across the globe. Medical facilities who specialize in geriatric care have reported success with the use of traditional Korean medicine not only in their Korean patients but also among Caucasian Americans. Statistical analysis of experiments involving more traditional remedies including herbal supplements and acupuncture have found that a patient's mental state is more relaxed and their emotional well-being often improves after being treated with more traditional remedies rather than or in combination with Western medicines, in which case improved mental and emotional stability has been seen [10]. Traditional Korean medicine is a longstanding and integral part of Korean cultural heritage. Since 1989, traditional Korean medicine has been formally incorporated into the health system and used alongside Western medicine. Most (86%) Koreans have used traditional Korean medicine at some point in their lives. Traditional Korean medicine is seen as safe and effective, particularly by older Koreans [11]. An annual percentage of the population using traditional Korean medicine has remained steady at approximately 5%, according to National Health Insurance claims [12].

Despite the fact that the elderly are particularly vulnerable to chronic conditions and disabilities, previous work addressing health services utilization has generally been limited. Research [13-16] has shown that health-related factors are important determinants of older Koreans using health services utilization and health-related factors, rather than socio-economic factors, are the better predictors of the use of Western medical services or traditional Korean medicine. It is important to consider the health status of the elderly because of their susceptibility to chronic conditions, such as arthritis, hypertension and diabetes.

The purpose of this study is to describe the health status and health services utilization of older Koreans and examine the factors that are associated with effective health services utilization. The findings are based on data from the first wave of the Korean Longitudinal Study of Ageing (KLoSA) conducted by the Korea Labor Institute. It is anticipated the findings of this study will be of value in identifying the subgroup differences in utilization. The Aday-Andersen model is used to identify the subgroup differences in utilization. Aday and Andersen introduce “equitable distribution”, which is important for addressing the problem of access to care [15,16]. It would be useful to utilize the model to interpret the results of this study.

## Methods

### Sample

The present study focuses on cross-sectional analyses using the 2008 Korean Longitudinal Study of Ageing, which was conducted with Korean men and women aged 45 years or older. The sample for this study was 4040 individuals who indicated they were 65 years old or older. The survey design was a 2-stage stratified cluster sampling framed by the national population and housing census.

For the analysis, the sample was weighted to reflect the population of the nation. To account for design effects created by stratified multistage cluster sampling, weights and strata were used in the estimation.

### Measures

The demographic and socioeconomic measures included age, gender, marital status, education and income. The health measures of health status included questions on whether the subject considered his or her health to be very good, good, fair, poor or very poor, and whether a chronic condition had been a problem during the last 24 months. Health services utilization included physician visits and the use of traditional Korean medicine. For physician visits, the actual volume of use (number of visits) during the previous 24 months was gathered. For the use of traditional Korean medicine, the respondents were asked whether they had visited traditional Korean medical practitioners during the 24 months preceding the interview.

There was no information missing for any of the demographic, socioeconomic or need variables. For the logistic regression analyses, the study variables were recoded so as to indicate dichotomies. The first category for a variable was coded 1 and the reference category for it (after “vs”) was coded 0. Health status, which is one of the need factors, is not a well‐defined concept, even though it is widely used. The several measures of health status were all reported in an interview format, and range from the highly subjective (e.g. self‐evaluation) to the more objective (e.g. conditions checked by a nursing assistant) [13]. In this study, two measures of health status were respectively represented as a dichotomy, i.e. ‘poor’ (1) versus ‘fair+’ (0), and ‘one or more chronic conditions’ (1) versus ‘no conditions’ (0). In addition to the health status of the elderly, the utilization of health care services is closely related to demographic factors – in particular age and sex. As proxies for need, age and sex were respectively represented as a dichotomy, i.e. ‘80+’ (1) versus ‘65-79’ (0) and ‘male’ (1) versus ‘female’ (0) in this analysis. Marital status was used and was represented by the binary variable ‘unmarried, divorced or widowed’ (1) versus ‘married’ (0). A categorical factor indicating the level of education attained is used as a supplementary measure of socio‐economic status. In this study, education was represented by the binary factor ‘primary school graduate’ (1) versus ‘middle school graduate +’ (0). As personal resources relate to service use, income was included. In the present study, income refers to the total individual income. The total annual income before deductions were reported was, ‘≤5,000,000 won (USD 4,400) to ‘**>**5,000,000 won’. Income was represented by the dichotomous factor ‘≤5,000,000 won’ (1) versus ‘**>**5,000,000 won’ (0).

### Data analysis

Bivariate analyses (chi-square tests) were used to examine differences between the men and women. Logistic regression analyses were then used to determine the factors significantly associated with health services utilization. The dependent variables in the logistic regression models included physician visits and the use of traditional Korean medicine. The independent variables were age, gender, education, marital status, income and the presence of chronic conditions. To avoid over-fitting the model, only those variables found to be significant on univariate analysis were included in the final regression model. All tests were conducted at the 5% level of significance. The percentage and the odds ratio (OR) were reported with a 95% confidence interval (CI).

## Results

The demographic and socioeconomic characteristics along with health services utilization are presented in Table 1. Survey respondents had a higher percentage with a lower education, a higher percentage with an income of 5 million won or less and a higher percentage who were younger, female, unmarried women, and married men (Table 1). The average age of the respondents was 73.5 ± 6.5 years old. The age of the respondents ranged from 65 to 107 years, and the average age for both men and women was 73.5 years old. There were statistically significant differences between men and women (*p* < 0.01). More men (5.7%) had at least a college education compared to women (0.8%) while more women (49.5%) than men (19.7%) had only a primary school education (*p* < 0.01). A larger number of men (37.5%) than women (27.5%) were married (*p* < 0.01). More men (13.1%) had an annual income of more than 5 million won compared to women (4.3%), while more women (46.7%) than men (20.5%) had an annual income of ≤5 million won (*p* < 0.01). The monthly average income respondents earned was 6,177,000 ± 9,240,000 won. 42.4% of the women and 27.3% of the men reported seeing a doctor in the past 2 years. A statistically significant number of women saw physicians more frequently than men (*p* < 0.01). The average number of physician visits was 12.3 ± 26.0. More women (16.9%) than men (6.7%) had been to see a traditional Korean medical practitioner (*p* < 0.01). The mean number of traditional Korean medical practitioner visits reported by both men and women was 3.6(±15.6).

**Table 1:** **Socioeconomic and health service use characteristics of the sample (N = 4,400)**

**Variable**	**Categories**	**Female**		**Male**		**Mean (±SD)**	**p-value**
		**N**	**%**	**N**	**%**		
Age (years)	65-69	738	18.3	561	13.9	73.5(±6.5)	< 0.01
	70-74	652	16.1	554	13.7		
	75-79	481	11.9	314	7.8		
	80+	485	12.0	255	6.3		
Education (graduation)	Primary school	2,000	49.5	798	19.7		< 0.01
	Middle school	205	5.1	286	7.1		
	High school	120	3.0	371	3.2		
	College+	31	0.8	229	5.7		
Marital status	Unmarried/divorced	1,110	30.8	1,515	4.2		< 0.01
	Married	1,246	27.5	169	37.5		
Income^#^	≤0-5,000 ,000 won	1,888	46.7	830	20.5	6,177,000(±9,240.000)	< 0.01
	5,000,001-7,500,000 won	192	4.7	180	4.5		
	7,500,001-10,000,000 won	101	2.5	146	3.6		
	>10,000,00 won	175	4.3	528	13.1		
Health care services	Traditional Korean medicine	684	16.9	269	6.7	3.6(±15.6)	< 0.01
	Physician visit	1,713	42.4	1,104	27.3	12.3(±26.0)	< 0.01

^#^Income in Korean monetary units.

Note: All other statistics not significant at p < 0.05.

### Self-rated health and chronic conditions

Table 2 presents the elders’ reports of self-rated health and chronic conditions. More women (29.3%) than men (14.5%) rated their health as poor or very poor (*p* < 0.01). The prevalence of self-reported chronic conditions was 23.0% (95% CI: 21.7-24.3%). There were statistically significant differences between men and women (*p* < 0.01). The prevalence of chronic conditions reported by women was 15.6% and men 7.4% (Table 2). The most common chronic conditions reported by women were uracratia (13.8%), arthritis (5.9%), hypertension (5.7%) and diabetes (1.7%). The most common chronic conditions reported by men were prostate-related (3.7%), hypertension (3.5%), arthritis (1.6%) and diabetes (1.1%). A significantly higher number of women than men reported having hypertension (*p* < 0.01), arthritis (*p* < 0.01) and heart disease (*p* < 0.01), while a larger number of men reported having cancer (*p* < 0.01) and lung problems (*p* < 0.01).

**Table 2:** **Health status and chronic conditions of the sample (N = 4,400)**

	**Female**		**Male**		**p-value**
	**N**	**%**	**N**	**%**	
Health status					< 0.01
Poor/very poor	1,182	29.3	589	14.5	
Fair	874	21.6	681	16.9	
Good/very good	300	7.4	414	10.2	
Presence of chronic diseases					< 0. 01
Yes	629	15.6	301	7.4	
No	1,727	42.7	1,383	34.2	
Chronic conditions					
Hypertension	140	5.7	86	3.5	< 0.05
Stroke	23	0.6	32	0.8	< 0.01
Heart disease	43	1.1	28	0.8	
Arthritis	178	5.9	50	1.6	< 0.01
Diabetes	57	1.7	38	1.1	
Lung problems	10	0.3	20	0.5	< 0.01
Liver problems	7	0.2	10	0.2	
Mental problems	28	0.7	13	0.3	
Cancer	13	0.3	28	0.7	< 0.01
Uracratia	262	13.8	-	-	
Prostate	-	-	57	3.7	

Note: All other statistics not significant at p < 0.05.

Multivariate logistic regression was performed to identify the significant predictors of respondents’ self-rated health. Self-rated poor health was higher among respondents of older age, who had only a primary education, who had chronic conditions and respondents with a personal income of ≤5 million won. Age, education, income and the presence of chronic conditions significantly predicted the residents’ self-rated poor health. Respondents with chronic conditions were twice as likely to report self-rated poor health (Odds Ratio: 2.26, 95% CI: 1.91-2.67) than residents with no chronic conditions (Table 3).

**Table 3:** **Multivariate logistic regression analysis of predictors of health services utilization for older Koreans, weighted (N = 4,400)**

**Variable**	**Poor health**		**Physician visit**		**Traditional Korean medicine**	
	**OR**	**95% CI**	**OR**	**95% CI**	**OR**	**95% CI**
Age	1.35**	1.11-1.63	1.40**	1.16-1.70	1.93**	1.52-2.46
80+						
vs. 65-79						
Sex			1.51**	1.26-1.81	2.15**	1.76-2.64
Male vs.						
Female						
Marital status						
Others vs.						
Married						
Education	1.82**	1.54-2.16				
≤Primary school						
vs. Middle school+						
Income	1.71**	1.46-2.01	1.39**	1.17-1.65	1.20*	1.00-1.43
≤5,0000,000 won						
vs. >5,000,000 won						
Health status			1.19*	1.02-1.38	1.55**	1.31-1.83
Poor						
vs. Fair+						
Chronic conditions	2.26**	1.91-2.67	1.55**	1.29-1.87	1.24*	1.03-1.50
Yes						
vs. No						
Model chi-square	**439,703.9**		**122,723.9**		**240,634.1**	
Degree of freedom	**6**		**7**		**7**	
Significance	**<0.0001**		**<0.0001**		**<0.0001**	

*P < 0.05.

**p < 0.01.

Note: All other statistics not significant at p < 0.05.

### Predictors of health services utilization

As presented in Table 3, the regression models were significant (p < 0.001) in predicting physician and traditional Korean medicine utilization by elderly Koreans. The odds ratios for health services utilization, simultaneously adjusted for multiple independent variables, are presented in Table 3. Those who were more likely to have used primary care services such as physicians and traditional Korean medicine included those 80 and older, men, those who had chronic condition or poor health and lower-income individuals. Age, sex, health status, chronic conditions, and income were important determinants of physician and traditional Korean medicine utilization among older Koreans.

## Discussion

The findings of this study provide important information on the health status and predictors of health services utilization among older Koreans. Although there were no statistically significant differences in self-rated poor health among men and women in the logistic regression analysis, this study indicated that more women (29.3%) than men (14.5%) rated their health as poor or very poor. This may be explained by the fact that women who grew up in traditional patriarchal families may have been socialized to place the needs of other family members before their own, which may have hindered them from seeking medical services [17]. The result of this study supports this explanation, i.e., older Korean women were less likely than their counterparts to have used physician and/or traditional Korean medical practitioner services (Table 3). More specifically, men were much more likely to have visited a doctor than women. They were also two times more likely to have used traditional Korean medicine than women. Health status was lower among those who had lower income, those who had primary education and persons of older age. These findings support other research [18,19] that demonstrates the importance of socio-economic status in self-reported poor health. The study by Laroche [18] shows that age seems to be a significant explanatory variable, suggesting that health deteriorates as one gets older. The study by Lim et al. [19] indicates that lower incomes and educational attainment are linked to bad health. Respondents with chronic conditions were twice as likely to have self-rated poor health as residents without a chronic condition. This result is consistent with the study that found a relationship between self-reported poor health and chronic conditions [20].

This study shows that health-related need factors, proxies for need (age, sex) and income determine health services utilization. Age, sex, income, health status, and chronic conditions predicted the use of primary care services, such as physician and traditional Korean medicine. Unlike previous studies [14-16], however, income is a significant predictor of physician and traditional Korean medicine utilization. Korean elders with lower incomes were more likely to consult a doctor or use traditional Korean medicine than those with higher incomes. Income had significant impact on the subgroup differences in the use of physician and traditional Korean medicine. Possible reasons for the different results may be that the study population is older or older people of lower income are simply in worse health than those of higher income. This is particularly an issue in the older population (especially those over 80). Similar concerns about higher service utilization have been born out in the medical aid program, which subsidizes health insurance co-payments for the low-income population in Korea [21]. Moreover, such higher use of service by the subsidized older adults is possible as the Korean health care system has no gate-keeping or care management system. The government recently introduced a care management program to monitor and guide medical aid beneficiaries with a high utilization of health care [22], but its effectiveness is still under evaluation.

These findings are subject to the limitations of secondary data. First, the model was limited to the data collected by Korean Labor Institute in 2008. The Korea Longitudinal Study of Ageing is a rich dataset. It focuses on the economic and social activities of the middle/old-age population, more than health status and health services utilization. The research identified few significant predictors of health status and health services utilization, from the array of factors in the model. Second, the data relies on subjective measures of health status and does not include objective measures of chronic diseases. Third, the long (two-year) recall period used to ask about health services utilization can increase the amount of bias associated with respondent memory loss. Although the U.S. National Center for Health Statistics National Health Interview Survey (NCHS-NHIS) uses a two-week recall period, it is a study that is continuously in the field throughout the year, and the data are used to construct aggregate estimates of the volume of visits for the U.S. population [23]. In contrast, the Korean Longitudinal Study of Ageing uses a two-year recall period and the data are used to construct the volume of visits estimates for individuals. Therefore, seasonal bias and random errors are more likely to be problematic with the latter approach.

## Conclusions

Respondents with poor health status were significantly older, less-educated, poorer and had a higher rate of chronic conditions. The study findings that higher numbers of older Korean women report poor health, point to the importance of providing culturally-appropriate health education programs that are tailored to the needs of older Korean women. Programs are needed that emphasize the importance of self-care and seeking early treatment. As this study shows with the finding of the high prevalence of uracratia, arthritis and hypertension, self-care and early screenings are very important for this population. Furthermore, prompt public measures are requested to control chronic conditions since they are associated with worsening health of Korean elders. It is also important to note that both health-related need factors and income had predictive power with regard to the use of physicians and traditional Korean medicine. This study provides an important contribution to the knowledge base of Korean elders. The findings show that elders in poor health status were significantly older and poorer, with higher rates of chronic conditions and health services utilization, which should help in the health care planning required to address this issue.

## Ethical statement

The present study focuses on cross-sectional analyses using the 2008 Korean Longitudinal Study of Ageing, which was conducted with Korean men and women aged 45 years or older. The Korean Longitudinal Study of Ageing was approved by the Research Ethics Committee of the Korea Labor Institute. The sample for this study was 4040 individuals who indicated they were 65 years old or older. The survey design was a 2-stage stratified cluster sampling framed by the national population and housing census.

## Consent

Written informed consent was obtained from the patient for the publication of this report.
